# Feasibility of Attachable Ring Stimulator for Intraoperative Neuromonitoring during Thyroid Surgery

**DOI:** 10.1155/2020/5280939

**Published:** 2020-01-30

**Authors:** Jongjin Kim, Hyeon Jong Moon, Young Jun Chai, Jung-Man Lee, Ki-Tae Hwang, Che-Wei Wu, Gianlorenzo Dionigi, Hoon Yub Kim, Kyung Sik Park, Sang Wan Kim, Ka Hee Yi

**Affiliations:** ^1^Department of Surgery, Seoul Metropolitan Government-Seoul National University Boramae Medical Center, Seoul 07061, Republic of Korea; ^2^Department of Thoracic and Cardiovascular Surgery, Seoul Metropolitan Government-Seoul National University Boramae Medical Center, Seoul 07061, Republic of Korea; ^3^Department of Anesthesiology and Pain Medicine, Seoul Metropolitan Government-Seoul National University Boramae Medical Center, Seoul 07061, Republic of Korea; ^4^Department of Otorhinolaryngology–Head and Neck Surgery, Kaohsiung Medical University Hospital, Kaohsiung Medical University, Kaohsiung, Taiwan; ^5^Division for Endocrine and Minimally Invasive Surgery, Department of Human Pathology in Adulthood and Childhood “G. Barresi”, University Hospital “G. Martino”, University of Messina, Messina, Italy; ^6^Department of Surgery, KUMC Thyroid Center, Korea University Anam Hospital, Korea University College of Medicine, Seoul 02841, Republic of Korea; ^7^Department of Surgery, Konkuk University School of Medicine, Seoul 05030, Republic of Korea; ^8^Department of Internal Medicine, Seoul National University College of Medicine and Seoul Metropolitan Government-Seoul National University Boramae Medical Center, Seoul 07061, Republic of Korea

## Abstract

**Objective:**

Stimulator-attached dissecting instruments are useful for intraoperative nerve monitoring during thyroidectomy. The aim of this study was to evaluate the feasibility of an attachable ring stimulator (ARS) by comparing the electromyography (EMG) amplitudes evoked by an ARS and a conventional stimulator.

**Methods:**

Medical records of fourteen patients who underwent thyroidectomy using intraoperative neuromonitoring between June and August 2019 were retrospectively reviewed. The amplitudes of V1, R1, R2, and V2 signals were checked using both the ARS and a conventional stimulator, at the same point.

**Results:**

Both stimulators were tested on 20 recurrent laryngeal nerves (RLNs) and 20 vagus nerves (VNs). In all the nerves, the amplitudes of V1, R1, R2, and V2 were greater than 500 *μ*V. The mean amplitudes of V1, R1, R2, and V2 checked with the ARS were 1175, 1432, 1598, and 1279 *μ*V, respectively. The mean amplitudes of V1, R1, R2, and V2 checked with the conventional stimulator were 1140, 1425, 1557, and 1217 *μ*V, respectively. Difference between amplitudes evoked by the two stimulators for V1, R1, R2, and V2 was 77, 110, 102, and 99 *μ*V, respectively. There was no statistical difference in the amplitudes between the two groups for V1, R1, R2, and V2.

**Conclusion:**

The ARS transferred electric stimulation as effectively as the conventional stimulator. It is an effective tool for repeated stimulation and facilitates continuous feedback regarding the functional integrity of nerves during thyroid surgery.

## 1. Introduction

One of the most common surgical complications of thyroid surgery is recurrent laryngeal nerve (RLN) injury. The incidence of transient palsy is reported to be as high as 5.2% and as high as 3.6% for permanent palsy [1, 2]. Although identification of the RLN is a routine and safe procedure, it is often difficult to evaluate the intactness of the RLN by naked eye visualization [3]. Therefore, since 1970, in bilateral procedures, it has been recommended that the contralateral lobe should not be resected until the integrity of the RLN has been confirmed by stimulation with an electrical current [4]. Nowadays, intraoperative neuromonitoring (IONM) is widely employed in thyroid surgery to monitor the functional intactness of the RLN and vagus nerve (VN).

In thyroid surgery, RLN injury occurs most frequently during traction of the thyroid gland or dissection of the soft tissue surrounding the RLN. Excessive traction on the thyroid can result in stretch injury of the RLN by Berry's ligament. Using energy-based devices for soft tissue dissection can cause thermal injury to the RLN [5, 6]. Using intermittent IONM (I-IONM), nerve function can be evaluated only when the nerve is stimulated, but not during surgical maneuvers between stimulations. Therefore, nerve injury cannot be avoided because nerve injury can only be detected after the injury has occurred.

**Figure 1:**
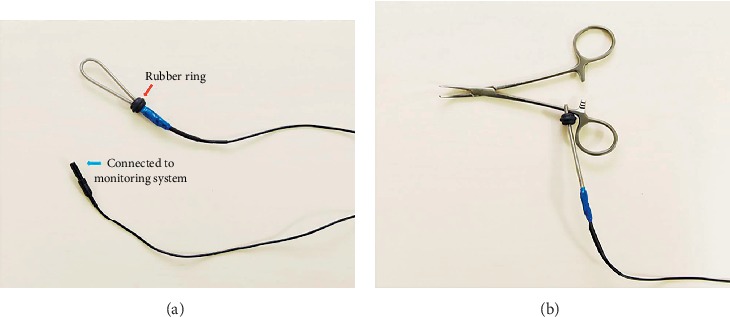
An attachable ring stimulator with a rubber ring for tightening (red arrow) (a). An attachable ring stimulator attached to mosquito forceps (b).

**Figure 2:**
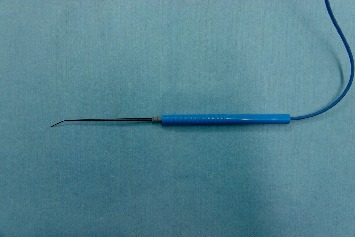
Conventional stimulator.

Recently, we adopted the use of an attachable ring stimulator (ARS; product name: stimulating ring electrode; product number: RSE1000; Medtronic, FL, Figure 1). During RLN dissection, the ARS was connected to mosquito forceps and continuously delivered an electric current to the RLN. The aim of the study was to evaluate the feasibility of the ARS by comparing the electromyography (EMG) amplitude of the vocal cord evoked by both the conventional stimulator (Figure 2, Prass Standard Monopolar Stimulator Probe, Medtronic, FL) and the ARS.

## 2. Materials and Methods

### 2.1. Patients

The medical records of patients who underwent thyroidectomy using I-IONM between June and August 2019 at Seoul Metropolitan Government-Seoul National University Boramae Medical Center were retrospectively reviewed. Surgery was performed by a single surgeon (Y. J. C). The institutional review board at Seoul Metropolitan Government-Seoul National University Boramae Medical Center approved this study (IRB no. L-2019-132).

### 2.2. Anesthesiology and Monitoring Setup

**Figure 3:**
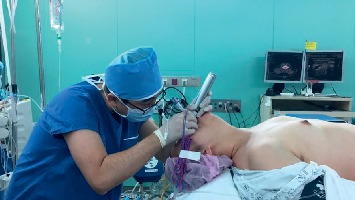
Prior to intubation, a pillow was placed beneath the neck for neck extension to avoid tube displacement during patient positioning.

Anesthesia was induced with glycopyrrolate (0.2 mg), lidocaine (30 mg), propofol (1.5 mg/kg), and fentanyl (100 *μ*g). After confirming loss of consciousness, rocuronium (0.3 ≥ 0.6 mg/kg) was administered for muscle relaxation. A pillow was placed beneath the neck for neck extension, prior to intubation, to avoid tube displacement during patient positioning. Then, a NIM^®^ EMG endotracheal tube (Medtronic, FL) was inserted for IONM (Figure 3). The electrode on the EMG endotracheal tube was positioned 1.5 cm below the arytenoid cartilage so that the electrode would be situated at the level of the vocal cord. Sugammadex (1 mg/kg) was administered to reverse the neuromuscular blockage effect of rocuronium. Anesthesia was maintained with a continuous infusion of propofol and remifentanil using a target-controlled infusion system. All anesthetic procedures were performed or supervised by a single anesthesiologist (J. L).

All setup and monitoring were performed in compliance with the standards outlined in the International Neural Monitoring Study Group (INMSG) guidelines [7]. Stimulation duration was set at 100 ms, the event threshold at 100 mV, and the stimulus current at 1 mA, with a frequency of 4 Hz. The RLN was considered to be successfully stimulated when the EMG amplitude was above 500 *μ*V during stimulation. Troubleshooting algorithms provided in the INMSG guideline 2018 were applied if the EMG amplitude was below 500 *μ*V [8]. For each surgery, the largest EMG amplitudes during stimulation were recorded.

### 2.3. Intraoperative Neuromonitoring Procedures

All patients underwent indirect laryngoscopic vocal cord examination and evaluation before and after thyroid lobectomy. During surgery, the ARS was attached to mosquito forceps for nerve stimulation. According to IONM guidelines, signals were recorded as follows: EMG amplitudes of the V1 (VN signal before surgical dissection), R1 (RLN signal at initial identification), R2 (RLN signal after thyroid removal and hemostasis), and V2 (VN signal after thyroid removal and hemostasis) [9]. In each patient, the carotid sheath was opened and the VN was fully exposed before testing V1 and the RLN was also fully exposed before testing R1. After the thyroid gland removal, the EMG amplitudes of the R2 and V2 signals were checked. V1, R1, R2, and V2 were checked using both the ARS and the conventional stimulator, at the same point, to compare the amplitudes evoked by each of the stimulators.

### 2.4. Statistics

Statistical analysis was performed using IBM SPSS Statistics for Windows, Version 20 (Armonk, NY: IBM Corp.). For continuous variables, data were expressed as mean ± standard deviation, and Student's *t*-test was used to compare the data between groups.

## 3. Results

Fifteen patients (1 males, 14 females) were included in this study (Table 1). Both an ARS and a conventional stimulator were tested on 40 nerves (20 RLN, 20 VN). The mean age of the patients was 48.1 ± 14.9 years. Mean tumor size was 1.5 ± 0.8 cm. Ten patients underwent thyroid lobectomy, and five patients underwent total thyroidectomy. No patient had vocal cord palsy on postoperative indirect laryngoscopic examination. Intraoperative neuromonitoring was successfully conducted in all patients, without any technical failure.

Table 2 demonstrates the EMG amplitude profiles of the RLN and VN stimulated by the ARS and the conventional stimulator. The mean amplitudes evoked by the ARS and the conventional stimulator were 1175 ± 658 *μ*V and 1140 ± 650 *μ*V for the V1, 1432 ± 789 *μ*V and 1425 ± 763 *μ*V for the R1, 1598 ± 828 *μ*V and 1557 ± 830 *μ*V for the R2, and 1279 ± 716 *μ*V and 1217 ± 679 *μ*V for the V2 signal. There was no statistical difference in the amplitudes between the two groups for V1 (*p*=0.867), R1 (*p*=0.979), R2 (*p*=0.876), and V2 (*p*=0.782). The mean difference between the amplitudes evoked by the two stimulators for V1, R1, R2, and V2 was 77 ± 114 *μ*V (range 5 to 530), 110 ± 152 *μ*V (range 2 to 670), 102 ± 103 *μ*V (range 6 to 3639), and 99 ± 79 *μ*V (range 1 to 372), respectively.

## 4. Discussion

Intermittent IONM is a significant modality for monitoring RLN and VN functions during thyroid surgery, and it predicts postoperative vocal cord function well. However, there is no evidence that the use of I-IONM reduces the incidence of RLN injury during thyroid surgery. One possible explanation for this is that I-IONM does not provide real-time feedback about RLN or VN function during surgical maneuvers [10]. On the other hand, C-IONM is an ideal modality to monitor the intactness of the RLN and the VN because it offers real-time feedback to surgeons. In a C-IONM system, continuous automatic stimulation is applied to the VN, and the EMG signal evoked by the vocal cord is monitored via electrodes on the endotracheal tube [11]. This facilitates the early detection of adverse EMG changes and alerts surgeons of the need to suspend surgical maneuvers immediately, thus preventing nerve injury [12, 13]. However, C-IONM has several limitations. First, additional procedures are required to use this device: the carotid sheath should be dissected, the VN should be fully exposed, the VN should be lifted, and the stimulating device should be applied on the VN. Second, continuous VN stimulation can cause hemodynamic instability, although the risk is low [14]. Last, C-IONM is costly because a device which delivers electric current to the VN is required. Considering the low incidence of RLN palsy, C-IONM cannot be used in all thyroid surgery cases because it may not be cost-effective [15].

In order to continuously monitor the RLN during thyroid surgery, without the use of C-IONM devices, stimulators have been connected to surgical instruments used near the RLN, including dissecting forceps and an energy-based device [14, 16, 17]. Studies reported that the EMG amplitudes evoked by this approach were comparable with those of a conventional stimulator. Another recent study introduced a detachable magnetic stimulator which could be attached to any metallic surgical instrument. This study also reported comparable nerve stimulation amplitudes between the detachable magnetic stimulator and a conventional stimulator [18].

The ARS is advantageous during the thyroid surgery in terms of time saving. Although it still takes time to move the ARS from one instrument to the other when the surgeon wants to change dissecting surgical instruments, alternating between the nerve stimulator and the dissecting instrument is more time-consuming and cumbersome compared to ARS. If the dissecting instrument is connected to the stimulator, the surgeon can stimulate the RLN with the dissecting instrument more frequently, and the time interval between stimulations is reduced. When the RLN is located in an unexpected position and the risk of RLN injury is particularly high, such as with nonrecurrent or bifurcated RLN [19, 20], a stimulator-connected dissector is helpful to locate the RLN because the EMG signal can be detected during exploration, before visual identification. In addition, stretch injury during soft tissue dissection may be avoided because the RLN is monitored continuously while the soft tissue covering the RLN is dissected. If the RLN is overstretched during dissection, the EMG amplitude decreases below threshold, which alerts the surgeon of the pending RLN injury so that surgical maneuvers may be suspended. Likewise, if an energy-based device is applied to the soft tissue while spreading the soft tissue with the dissecting instruments, the thermal effect of the energy-based device on the RLN can be monitored.

In this study, the amplitudes evoked by the ARS and the conventional stimulator were comparable. In addition, there are additional advantages related to the ARS used in the current study. The ARS can be easily attached to most surgical instruments (such as mosquito forceps, tonsil forceps, Mixter forceps, and Metzenbaum scissors) by tightening a rubber ring. The ARS was developed to monitor nerves originating from the spinal cord in spinal surgery and has been used following approval from the regulatory administrative body. Unlike other attachable stimulators, which cannot be used without approval from the local medical administrative body, the ARS used in this study is already commercialized and can be used without regulatory approval. As a disadvantage, stimulus cannot be delivered to the nerve if the rubber ring becomes loosened from the surgical instrument. Therefore, the tightness of the rubber ring should be closely monitored.

The most common reason for IONM failure is malposition of the endotracheal tube [7]. In fact, upward or downward migration of the endotracheal tube by 1 cm can cause a significant decrease in EMG amplitude [21, 22]. During patient positioning, tube migration more than 1 cm occurs in 12.7% of the patients [23] and tube repositioning is required in 5% of patients [24]. In this study, prior to intubation, a pillow was placed beneath the patient's neck for neck extension to avoid tube displacement during patient positioning. In the current study, the EMG amplitudes of the RLN and VN were above 500 *μ*V.

There are limitations in the use of the ARS. First, the instruments used in this study had no insulation coating which resulted in electrical current shunting and no EMG response if the shaft of the instrument was in contact with skin or soft tissue while the surgeon stimulated the RLN with the tip of the instrument. Therefore, the surgeon used a high-stimulus current (2 or 3 mA) before visualizing the RLN and had to avoid touching the surrounding tissue during nerve stimulation. We propose that insulated instruments with the tip being only exposed should be developed for more effective stimulation. Second, the ARS basically facilitates I-IONM, although the surgeon can stimulate the RLN continuously around the RLN using the ARS and receive continuous feedback. The ARS only gives information about the integrity of the RLN distal to the stimulation point, and the integrity of the proximity to the stimulation point cannot be assessed. In addition, the amplitude may alter depending on the conditions such as the dissector's contacting area with the nerve or contact duration. Therefore, C-IONM, which monitors RLN integrity along its whole course of the neck by stimulating the VN is more effective than the ARS in terms of real-time evaluation of the traction injury. In addition, latency changes as a component of EMG changes in case of impending nerve injury cannot be determined with the ARS. Thus, the ARS may be inferior to C-IONM in terms of preventing tractional nerve injury.

## 5. Conclusions

This study demonstrated that ARS can be safely used for IONM during thyroid surgery and that the ARS transferred electric stimulation as effectively as a conventional stimulator. The ARS may be used to obtain real-time feedback about the functional status of the RLN during thyroid surgery.

## Figures and Tables

**Table 1:** Clinicopathological characteristics and surgical outcomes of the patients.

Variables	Values
Gender (male : female)	1 : 14
Age (mean ± SD), years	48.1 ± 14.9
Tumor size (mean ± SD), cm	1.5 ± 0.8
Pathology	
Papillary thyroid carcinoma	13
Follicular adenoma	2
Operative extent	
Lobectomy	10
Total thyroidectomy	5
Vocal cord palsy	0

MRND; modified radical neck dissection.

**Table 2:** EMG amplitude profiles of recurrent laryngeal nerve and vagus nerve stimulated by the attachable ring stimulator and conventional stimulator.

Patient no.	Side	V1 (*μ*V)			R1 (*μ*V)			R2 (*μ*V)			V2 (*μ*V)		
		Ring	Conventional	Diif^*∗*^	Ring	Conventional	Diif^*∗*^	Ring	Conventional	Diif^*∗*^	Ring	Conventional	Diif^*∗*^
1	Rt	2177	2077	100	2572	2406	166	2361	2460	99	2266	2262	4
	Lt	1128	1177	49	1843	1733	110	2178	2530	352	1563	1191	372
2	Lt	1533	1003	530	1103	1773	670	1587	1635	48	996	1073	77
3	Lt	1735	1581	154	2599	2474	125	2658	2487	171	2182	2176	6
4	Lt	1021	1026	5	1375	1392	17	1216	1241	25	864	949	85
5	Rt	641	677	36	730	809	79	770	699	71	743	654	89
6	Rt	1031	977	54	2278	2188	90	1992	1799	193	1214	1111	103
7	Rt	3004	3043	39	2849	2809	40	2636	2583	53	2771	2539	232
	Lt	1561	1505	56	1469	1518	49	1741	1584	157	1636	1774	138
8	Lt	762	808	46	541	566	25	834	850	16	561	571	10
9	Rt	621	640	19	716	801	85	1460	1356	104	1219	1157	62
10	Rt	660	653	7	788	786	2	862	902	40	690	689	1
	Lt	1026	1058	32	956	981	25	650	603	47	668	634	34
11	Rt	2100	2159	59	2827	2735	92	3639	3633	6	2781	2595	186
12	Lt	607	628	21	1040	1038	2	2403	2040	363	1487	1255	232
13	Lt	849	697	152	1050	727	323	945	818	127	705	546	159
14	Rt	594	525	69	738	578	160	967	953	14	791	757	34
	Lt	1146	1195	49	1666	1746	80	1391	1356	35	1177	1084	93
15	Rt	760	792	32	730	693	37	879	784	95	720	768	48
	Lt	541	579	38	761	747	14	794	824	30	537	557	20
Mean		1175	1140	77	1432	1425	110	1598	1557	102	1279	1217	99
*p* value^†^				0.867			0.979			0.876			0.782

^∗^Difference between the amplitudes evoked by the attachable ring stimulator and conventional stimulator. ^†^*p* value for the mean amplitudes between the two groups.

## Data Availability

The data used to support the findings of this study are included within the article.
